# Prognostic Value of Immune-Related Genes in the Tumor Microenvironment of Bladder Cancer

**DOI:** 10.3389/fonc.2020.01302

**Published:** 2020-07-28

**Authors:** Faping Li, Haolin Teng, Mingdi Liu, Bin Liu, Difei Zhang, Zhixiang Xu, Yishu Wang, Honglan Zhou

**Affiliations:** ^1^Department of Urology, The First Hospital of Jilin University, Changchun, China; ^2^Key Laboratory of Pathobiology, Ministry of Education, Jilin University, Changchun, China

**Keywords:** bladder cancer, tumor microenvironment, prognosis, immune score, stromal score, overall survival, cancer-specific survival

## Abstract

The tumor microenvironment (TME) is a complex system that plays an important role in tumor development and progression, but the current knowledge about its effect on bladder cancer (BC) is scarce. In this study, we performed a comprehensive analysis of the relationship between the TME and gene expression profiles to identify prognostic biomarkers for BC. The ESTIMATE algorithm was used to calculate immune and stromal scores of BC patients who were obtained from the Gene Expression Omnibus database. We found that the immune and stromal scores were associated with clinical characteristics and the prognosis of BC patients. Based on these scores, 104 immune-related differentially expressed genes were identified. Further, functional enrichment analysis revealed that these genes were mainly involved in the immune-related biological processes and signaling pathways. Three prognostic genes were then identified and used to establish a risk prediction model using Cox regression analyses. Kaplan–Meier survival analysis showed that the expression levels of COL1A1, COMP, and SERPINE2 significantly correlated with cancer-specific survival and overall survival of BC patients. Additionally, we validated the prognostic values of these genes using two independent cohorts from The Cancer Genome Atlas and Gene Expression Omnibus databases. Finally, the relationships between the three prognostic genes and several immune cells were evaluated using Tumor Immune Estimation Resource, indicating that the expression levels of COL1A1, COMP, and SERPINE2 correlated positively with the tumor infiltration levels of CD4^+^ T cells and macrophages. In conclusion, the current study comprehensively analyzed the TME and presented immune-related prognostic genes for BC, providing new insights into immunotherapeutic strategies for BC patients.

## Introduction

Bladder cancer (BC) is the 10th most common malignancy worldwide with 549,000 new cases and 200,000 deaths in 2018 (1). It is a heterogeneous disease, which is divisible into non-muscle-invasive BC (NMIBC) and muscle-invasive BC (MIBC) based on the invasion of lamina propria (2). At initial diagnosis, 70–75% of patients are diagnosed as NMIBC while the remaining 25–30% suffer from MIBC (3). Unfortunately, it is estimated that 60–70% of NMIBCs will relapse and 10–30% of these cases will progress to an advanced crippling morphology despite seasonable intensive therapy (4). When the lesions are confined to the mucosal or sub-mucosal connective tissues, BC patients have a relatively favorable prognosis with a 5-year cancer-specific survival (CSS) rate ranging from 88.5 to 99.1% (5). However, once the tumor progresses to MIBC, the 5-year CSS rate decreases to 40% (6). The lack of understanding about the molecular mechanism behind BC development leads to the deficiency of effective therapy for BC, especially MIBC. Thus, it is still important to identify novel biomarkers for the early diagnosis and develop effective therapeutic strategies for BC patients.

Tumor progression is a complex process that depends on the heterogeneous components of the tumor microenvironment (TME), which includes stromal, endothelial, and tumor-infiltrating immune cells (7). Recently, the TME has increasingly become a compelling topic due to its significant impact on tumor progression, therapeutic resistance, and clinical outcomes. Tumor-infiltrating stromal and immune cells, which are two major components of the TME, have been deemed to be of potential value in the diagnosis and prognosis of cancer (8, 9). For example, infiltration of macrophages in BC was associated with T cell tolerance and could affect the outcome of patients with MIBC (10, 11). In addition, the CC-chemokine receptor (CCR8) has been identifed as an important marker expressed on intratumoral regulatory T cells (Tregs). Wang et al. (12) confirmed that the heavy CCR8^+^Tregs accumulation in MIBC was associated with the immune tolerance and contributed to chemotherapy resistance and poor prognosis of MIBC. Therefore, it is necessary to study the diverse components of the microenvironment and their complex communications for identifying novel therapeutic targets.

ESTIMATE algorithm is a newly developed method, which is used to predict the infiltration levels of non-tumor cells by analyzing their specific gene expression signature (13). Using this algorithm, stromal score and immune score can be generated based on the single sample Gene Set Enrichment Analysis. Previous studies have applied this algorithm to calculate the immune scores and stromal scores of BC samples to identify the TME-related genes with poor prognosis (14, 15). Besides, the ESTIMATE algorithm has been also applied to clear cell renal cell carcinoma (16), breast cancer (17), and lung cancer (18), confirming its feasibility and effectiveness.

In this study, we calculated immune and stromal scores of BC samples from the Gene Expression Omnibus (GEO) database using the ESTIMATE algorithm and correlated these scores to clinical characteristics and prognosis of BC patients. Based on these scores, the immune-related differentially expressed genes (DEGs) were identified. Then we applied Cox regression analyses to identify immune-related prognostic genes and establish prognostic gene signature. To further investigate the molecular and infiltrating immune cells in the TME, we evaluated the associations between the prognostic genes and several immune cells based on Tumor Immune Estimation Resource (TIMER). Moreover, we verified the availability and reliability of the prognostic genes using two independent cohorts from The Cancer Genome Atlas (TCGA) and GEO databases.

## Materials and Methods

### Patients and Dataset

The gene expression series matrix and clinical characteristics of BC patients were downloaded from the GEO dataset GSE13507 (https://www.ncbi.nlm.nih.gov/geo/). One hundred and sixty-five eligible patients with complete clinical information were included in this study. In terms of the gene expression profile, we downloaded the unprocessed raw data for calculating the immune and stromal scores. Both gene expression data and corresponding clinical characteristics were publicly available. Therefore, there was no requirement for ethics committee approval.

### Calculation of Immune and Stromal Scores

The ESTIMATE algorithm was applied to evaluate the infiltrating levels of the immune and stromal cells in the BC tissues. Based on the gene expression data, the immune and stromal scores were calculated using the “estimate” R package. Furthermore, the best cutoff values generated using X-tile plots (19) were used to divide the samples into high and low score groups. The clinical characteristics between the high and low score groups were compared using a chi-square test. The survival curves were drawn using GraphPad Prism 8 (GraphPad Software Inc., United States) and the log-rank test was employed to test the statistic difference with the significance level *p* < 0.05.

### Identification of DEGs and Functional Enrichment Analyses

The “limma” R package was applied to analyze the matrix data of gene expression levels for identifying DEGs between the high and low score groups. Log_2_ (fold change) >1 or < −1 and *p* < 0.05 were set as the threshold for filtering DEGs. Moreover, an immune-related gene list was obtained from the ImmPort platform (https://www.immport.org/) (20), and utilized to identify immune-related DEGs by the Venny online tool (Venny 2.1, https://bioinfogp.cnb.csic.es/tools/venny/). The DAVID database was then employed to perform the functional enrichment analyses of Gene Ontology (GO) and Kyoto Encyclopedia of Genes and Genomes (KEGG) pathway. *P* < 0.05 was considered to be statistical significant.

### Establishment of a Risk Prediction Model

Next, we established a risk model and identified the immune-related prognostic genes for predicting the prognosis of patients with BC. First, we assessed the relationships between the expression levels of the selected DEGs and CSS of patients by univariate Cox regression analysis using the “survival” R package. The significant genes with *p* < 0.05 were screened out for further analysis. Subsequently, using the “glmnet” R package, we performed the Least Absolute Shrinkage and Selector Operation (LASSO) analysis that could reduce the estimation variance while providing an explicable final model (21), and identified the key genes affecting patients' prognosis. Finally, we conducted a multivariate Cox regression analysis to establish a risk prediction model. The risk scores for each patient were generated using the Equation: Risk score = Exp_1_^*^ β_1_ + Exp_2_^*^ β_2_ + ···Exp_n_^*^ β_n_, where “Exp” represented the expression level of key gene while “β” is the regression coefficient acquired from the multivariate Cox regression analysis. The best cutoff value was generated using X-tile plots and was set as the threshold to divide patients into high- and low-risk groups.

### Evaluation of the Risk Prediction Model and Survival Analysis

Further, a receiver operating characteristic (ROC) curve was utilized to evaluate the efficiency of the risk prediction model. The ROC curves with the area under the curve (AUC) values were visualized using the “timeROC” package. Kaplan–Meier curves were drawn to exhibit the associations of risk score and potential prognostic genes with survivals of patients. The log-rank test was employed to test the statistic difference with the significance level *p* < 0.05.

### Validation of Prognostic Value

TCGA is a publicly collaborative project that includes lots of clinical information and gene expression profiles across 33 cancer types (22, 23). To confirm the reliability of the identified prognostic genes from the GEO data, BC data from TCGA were utilized to perform validation with the Kaplan–Meier Plotter (http://kmplot.com/analysis/) and Gene Expression Profiling Interactive Analysis (GEPIA) database (http://GEPIA.cancer-pku.cn/). In addition, BC data from the GEO dataset GSE32548 were also utilized to perform validation using the online survival analysis tool OSblca (http://bioinfo.henu.edu.cn/BLCA/BLCAList.jsp) (24).

### Analysis of Immune Cell Infiltration

TIMER is also a publicly available resource that can be used to assess the abundances of six infiltrating immune cells, including B cells, CD4^+^ T cells, CD8^+^ T cells, neutrophils, macrophages, and dendritic cells (DCs). We investigated the correlation between the prognostic genes in BC and the abundances of the six immune cells using the online tool TIMER (https://cistrome.shinyapps.io/timer/).

## Results

### Immune and Stromal Scores Correlate With BC Clinical Characteristics and Prognosis

**Figure 1:**
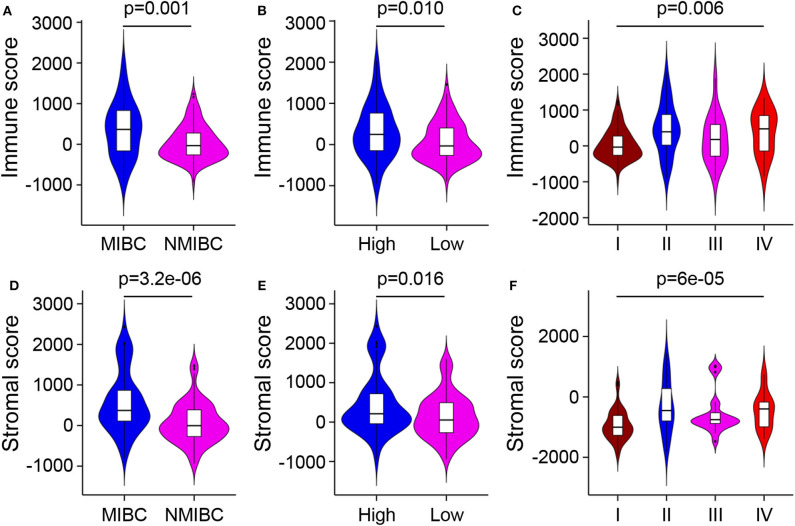
Relationship between immune and stromal scores and BC clinical characteristics. **(A–C)** Distributions of immune scores among different BC invasions, differentiation grades, and stages. **(D–F)** Distributions of stromal scores among different BC invasions, differentiation grades, and stages. MIBC, muscle-invasive bladder cancer; NMIBC, non-muscle-invasive bladder cancer.

Gene expression profiles and corresponding clinical information of 165 BC samples were obtained from the dataset GSE13507. After normalizing, 10,180 messenger RNAs were extracted from gene expression profiles. The immune and stromal scores of these samples were calculated by the ESTIMATE algorithm using the “estimate” R package (Supplementary Table 1). Using X-tile plots, the best cutoff value was generated to divide patients into high and low score groups. Table 1 displayed the different distributions of clinical characteristics with respect to scores. Further, the statistical analyses showed that both immune and stromal scores of MIBC, high-grade, and later stage BC were significantly higher compared to NMIBC, low-grade, and earlier stage groups (Figures 1A–F; *p* < 0.05). But the immune and stromal scores were not associated with BC recurrence (*p* = 0.13 and *p* = 0.22, respectively) and progression (*p* = 0.72 and *p* = 0.91, respectively) (Supplementary Figures 1A–D).

**Table 1:** Clinicopathologic characteristics of patients in different immune/stromal score groups.

**Variables (%)**	**Immune score**			**Stromal score**		
	**Low (*n* = 124)**	**High (*n* = 41)**	***p-*value**	**Low (*n* = 80)**	**High (*n* = 85)**	***p-*value**
Age (years)	65.4 (24–88)	64.6 (32–82)	0.725	65.3 (24–88)	65.1 (32–84)	0.913
Gender			0.010			0.066
Female	17 (13.7)	13 (31.7)		10 (12.5)	20 (23.5)	
Male	107 (86.3)	28 (68.3)		70 (87.5)	65 (76.5)	
Invasiveness			<0.001			<0.001
MIBC	36 (29.0)	26 (63.4)		15 (18.8)	47 (55.3)	
NMIBC	88 (71.0)	15 (36.6)		65 (81.2)	38 (45.7)	
Grade			0.023			0.014
Low	85 (68.5)	20 (48.8)		58 (72.5)	46 (54.1)	
High	39 (31.5)	21 (51.2)		22 (27.5)	39 (45.9)	
Recurrence			0.657			0.329
Yes	30 (24.2)	6 (14.6)		25 (31.3)	11 (12.9)	
No	58 (46.8)	9 (22.0)		40 (50.0)	27 (31.8)	
Progression			0.289			0.681
Yes	21 (16.9)	10 (24.4)		14 (17.5)	17 (20.0)	
No	103 (83.1)	31 (75.6)		66 (82.5)	68 (80.0)	
Stage			0.001			<0.001
I	88 (71.0)	15 (36.6)		65 (81.3)	38 (44.7)	
II	15 (12.1)	11 (26.8)		6 (7.5)	20 (23.5)	
III	13 (10.5)	7 (17.1)		4 (5.0)	16 (18.8)	
IV	8 (6.4)	8 (19.5)		5 (6.2)	11 (13.0)	

**Figure 2:**
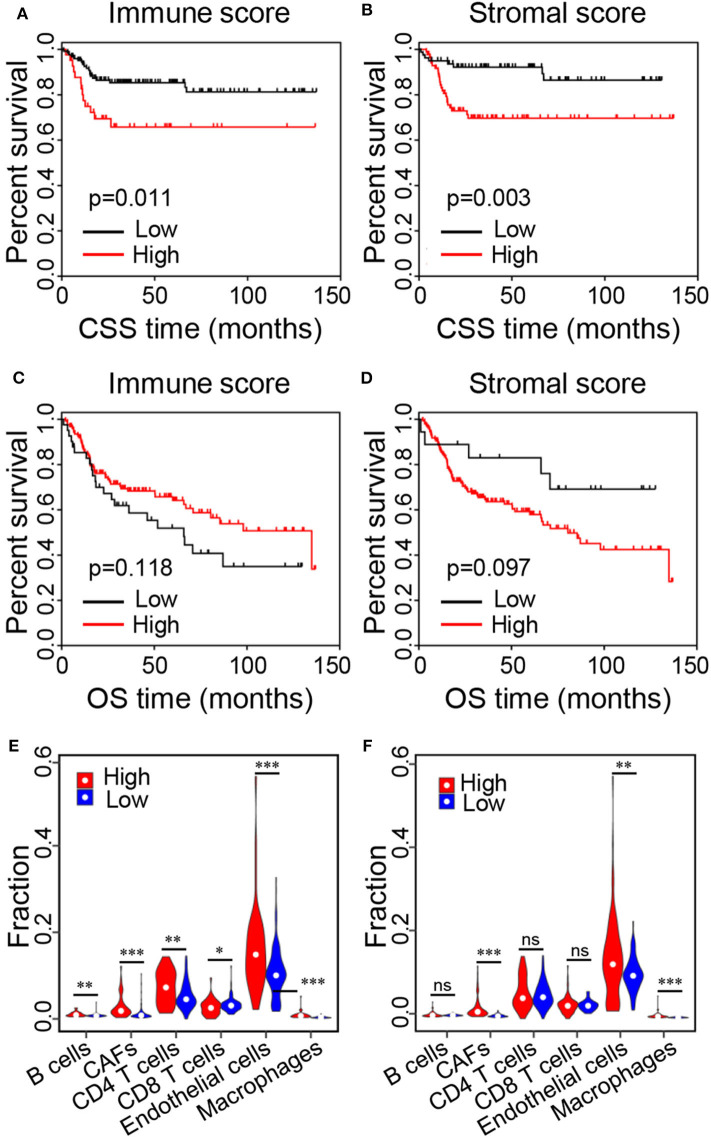
Kaplan–Meier survival curves of BC patients and relative proportions of immune cells in the high and low score groups. **(A,B)** Kaplan–Meier curves of CSS for patients with low vs. high immune and stromal scores. **(C,D)** Kaplan–Meier curves of OS for patients with low vs. high immune and stromal scores. **(D–F)** Relative proportions of immune cells in the low vs. high immune and stromal score groups (*p < 0.05, **p < 0.01, ***p < 0.001, ns, no significance). CSS, cancer-specific survival; OS, overall survival.

To explore whether the immune and stromal scores were related to CSS and overall survival (OS), BC patients were divided into two groups based on the best cutoff value generated using X-tile plots. Kaplan–Meier survival curves were performed to analyze the correlation, revealing that the high immune and stromal scores negatively correlated with CSS of BC patients (Figures 2A,B; *p* = 0.011 and 0.003, respectively). Whereas, no significant correlation was found between the immune/stromal scores and OS of BC patients (Figures 2C,D; *p* = 0.118 and 0.097, respectively).

To further analyze the reasons for the poor outcomes of the patients with higher scores, we assessed the proportions of immune cells in the TME of the high and low score groups using the online tool Estimate the Proportion of Immune and Cancer cells (EPIC) (25). Specifically, the proportions of B cells (*p* = 0.002), cancer-associated fibroblasts (CAFs, *p* = 1.2e-08), CD4^+^ T cells (*p* = 0.003), endothelial cells (*p* = 2.9e-04), and macrophages (*p* = 2.0e-12) were significantly higher in the high immune score group compared with the low immune score group, while the proportions of CD8^+^ T cells were significantly lower (Figure 2E; *p* = 0.024). In addition, the proportions of CAFs (*p* < 2.0e-16), endothelial cells (*p* = 0.005), and macrophages (*p* = 2.1e-13) were significantly higher in the high stromal score group compared with the low stromal score group (Figure 2F). These results indicated that high immune and stromal scores were related to adverse prognosis in BC patients.

### Identification of DEGs and Functional Enrichment Analyses

**Figure 3:**
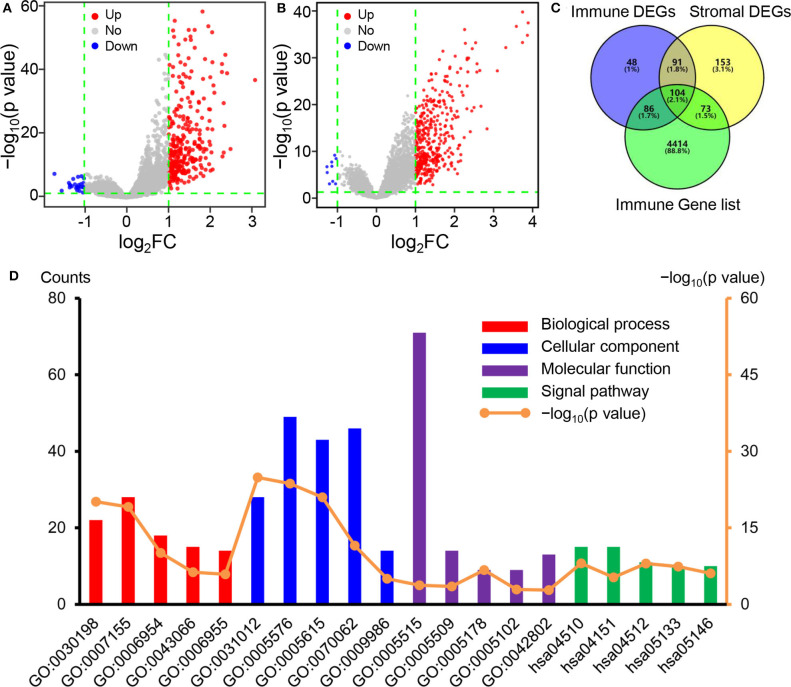
Identification of DEGs and functional enrichment analyses. **(A)** Volcano plot of DEGs based on immune score in BC samples. **(B)** Volcano plot of DEGs based on stromal score in BC samples. **(C)** Venn diagrams showing the overlapping genes among immune score-related DEGs, stromal score-related DEGs, and immune function-related genes. **(D)** The top five GO and KEGG pathway terms of the overlapping immune-related DEGs. DEGs, differentially expressed genes; BC, bladder cancer; GO, Gene Ontology; KEGG, Kyoto Encyclopedia of Genes and Genomes.

To explore the association of the gene expression levels with the stromal/immune scores, we compared the gene microarray data of all 165 samples obtained in the dataset GSE13507. Using “limma” package, we identified 329 genes with differential expression between the high and low immune score group. Similarly, 421 genes were identified by comparison of the high and low stromal score groups. The volcano plots showed that 304 and 412 genes were up-regulated in the high immune and stromal score groups, respectively (Figure 3A), while 25 and 9 genes were down-regulated (Figure 3B). Subsequently, we identified the overlapping genes among immune score-related DEGs, stromal score-related DEGs, and immune function-related genes obtained from the ImmPort database (Figure 3C). The 104 overlapping genes showed in the Venn diagrams were selected for further analysis.

Later, we performed functional enrichment analyses to comprehend the functional properties of the 104 differential immune-related genes. Using the DAVID tool, we identified 173 GO terms and 28 KEGG terms in which the immune-related DEGs enriched significantly (*p* < 0.05). Figure 3D showed the top five KEGG pathway terms and GO terms, including biological processes, cellular component, and molecular function. The top GO terms, including extracellular matrix organization, inflammatory response, and immune response (Table 2), were strongly associated with the immune microenvironment of tumors. Moreover, the KEGG pathways mainly focused on focal adhesion and PI3K-Akt signaling pathway (Table 2), which were also closely related to the immune microenvironment.

**Table 2:** Enrichment analysis of the 104 differential immune-related genes.

**Category**	**Term**	**Description**	**Count**	***p-*value**
BP	GO:0030198	ECM organization	22	7.63e-21
BP	GO:0007155	Cell adhesion	28	8.22e-20
BP	GO:0006954	Inflammatory response	18	8.64e-11
BP	GO:0043066	Negative regulation of apoptotic process	15	4.96e-07
BP	GO:0006955	Immune response	14	1.26e-06
CC	GO:0031012	ECM	28	1.27e-25
CC	GO:0005576	Extracellular region	49	2.06e-24
CC	GO:0005615	Extracellular space	43	9.99e-22
CC	GO:0070062	Extracellular exosome	46	2.95e-12
CC	GO:0009986	Cell surface	14	9.21e-06
MF	GO:0005178	Integrin binding	9	1.98e-07
MF	GO:0005515	Protein binding	71	1.88e-04
MF	GO:0005509	Calcium ion binding	14	3.09e-04
MF	GO:0005102	Receptor binding	9	1.18e-03
MF	GO:0042802	Identical protein binding	13	1.56e-03
KEGG	hsa04510	Focal adhesion	15	8.88e-09
KEGG	hsa04151	PI3K-Akt signaling pathway	15	5.10e-06
KEGG	hsa04512	ECM-receptor interaction	11	9.88e-09
KEGG	hsa05133	Pertussis	10	3.92e-08
KEGG	hsa05146	Amoebiasis	10	8.05e-07

*BP, biological process; CC, cellular component; MF, molecular function; KEGG, Kyoto Encyclopedia of Genes and Genomes; ECM, extracellular matrix; PI3K-Akt, phosphatidylinositol-3 kinase-protein kinase B*.

### Identification of Genes Associated With Prognosis and Establishment of the Risk Prediction Model

**Figure 4:**
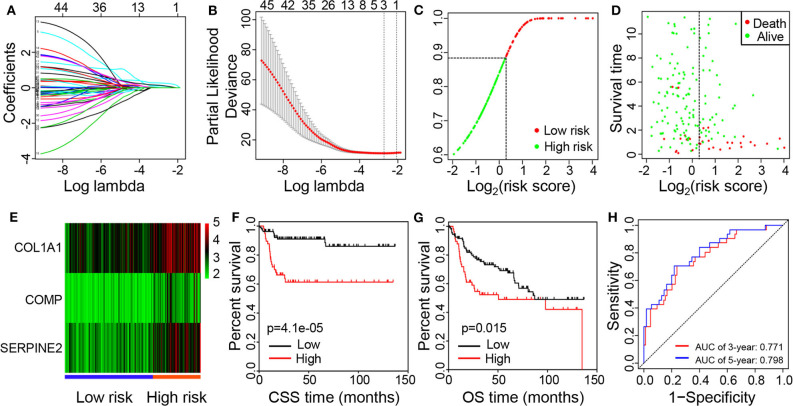
Identification of prognostic genes and establishment of the risk prediction model. **(A)** LASSO coefficient profiles of the 45 significant genes from the result of the univariate regression. **(B)** Feature selection for prognostic biomarkers using the LASSO method. **(C)** Distributions of risk score. **(D)** The survival time of patients in high- and low-risk groups. **(E)** Heat map of expression levels of the three prognostic genes in the high- and low-risk groups. **(F)** Kaplan–Meier curves of CSS for patients with low vs. high risk scores. **(G)** Kaplan–Meier curves of OS for patients with low vs. high risk scores. **(H)** ROC curves of the risk model for predicting 3- and 5-year survival rates. LASSO, Least Absolute Shrinkage and Selector Operation; CSS, cancer-specific survival; OS, overall survival; ROC, receiver operating characteristic.

To further explore the prognostic significance of the immune-related DEGs, we performed the Cox regression analyses. The univariate regression analysis revealed that 45 immune-related genes were significantly associated with CSS of BC patients (Supplementary Table 2). Then, the 45 significant genes from the result of the univariate regression were utilized for the multiple LASSO regression (Figure 4A), and three key genes were identified (Figure 4B). Finally, the risk prediction model was built with the three immune-related genes, namely, COL1A1, COMP, and SERPINE2.

Based on relative coefficients in the multivariate regression analysis, the risk scores were calculated according to the formula: (0.187 ^*^ COL1A1 expression level) + (0.293 ^*^ COMP expression level) + (0.243 ^*^ SERPINE2 expression level). 165 patients were divided into high- and low-risk groups according to the best cutoff value generated using X-tile plots (Figure 4C). The survival times of BC patients were significantly shorter while the number of deaths was higher in the high-risk group compared with the low-risk group (Figure 4D). Moreover, higher expression levels of COL1A1, COMP, and SERPINE2 were observed in the high-risk group compared with the low-risk group (Figure 4E). The Kaplan-Meier survival analysis revealed that a high-risk score was significantly associated with poor CSS (Figure 4F; *p* = 4.1e-05) and OS (Figure 4G; *p* = 0.015). As presented graphically in Figure 4H, according to the ROC curve, the AUC for the risk model in predicting 3- and 5-year survival rates were 0.771 and 0.798, respectively.

### Survival Analysis and Validation of the Prognostic Value

**Figure 5:**
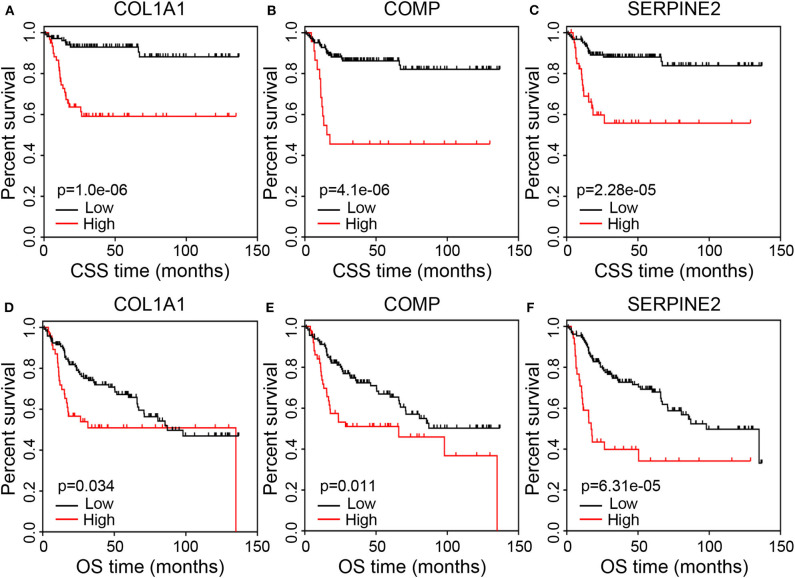
Correlation of the prognostic genes with survival of BC patients. **(A–C)** Kaplan–Meier curves of CSS for patients grouped by expression levels of COL1A1, COMP, and SERPINE2. **(D–F)** Kaplan–Meier curves of OS for patients grouped by expression levels of COL1A1, COMP, and SERPINE2. BC, bladder cancer; CSS, cancer-specific survival; OS, overall survival.

To further assess the potential prognostic value, we conducted survival analysis concerning the three immune-related genes and patients' prognosis. The results showed that the high expression levels of COL1A1, COMP, and SERPINE2 were associated with poor CSS (Figures 5A–C; *p* < 0.001). Similarly, the high expression levels of COL1A1 (*p* = 0.034), COMP (*p* = 0.011), and SERPINE2 (*p* = 6.31e-05) negatively correlated with OS (Figures 5D–F).

To verify whether the prognostic value of the three immune-related genes identified by GEO analyses was reliable and available in other cases of BC, we selected two independent cohorts of BC cases from the TCGA and GEO databases. Kaplan–Meier curves generated using the Kaplan-Meier Plotter database revealed that BC patients in the high COL1A1 (*p* = 0.003) and COMP (*p* = 0.003) expression groups suffered a significantly shorter OS than those in the low expression groups (Supplementary Figures 2A,B). BC patients in the high SERPINE2 expression group had shorter OS compared to the low expression group, although this did not reach a statistical significance (Supplementary Figure 2C; *p* = 0.066). The median OS was 28.8 months in the high expression group and 88.03 months in the low expression group. Furthermore, Kaplan–Meier curves generated using the GEPIA database revealed that the high expression levels of COMP and SERPINE2 were significantly associated with poor prognosis for OS (*p* = 0.005 and 8.1e-05, respectively; Supplementary Figures 2E,F). But there was no obvious relationship between the COL1A1 expression level and OS (Supplementary Figure 2D; *p* = 0.11). Finally, we used the OSblca tool to verify the prognostic value of the three genes. As shown in Supplementary Figures 3A–C, the high expression levels of COL1A1 (*p* = 6e-04), COMP (*p* = 0.026), and SERPINE2 (*p* = 0.013) negatively correlated with OS. These data suggested that COL1A1, COMP, and SERPINE2 could be valuable prognostic factors in BC.

### Correlations of the Prognostic Genes With Infiltrating Immune Cells

**Figure 6:**
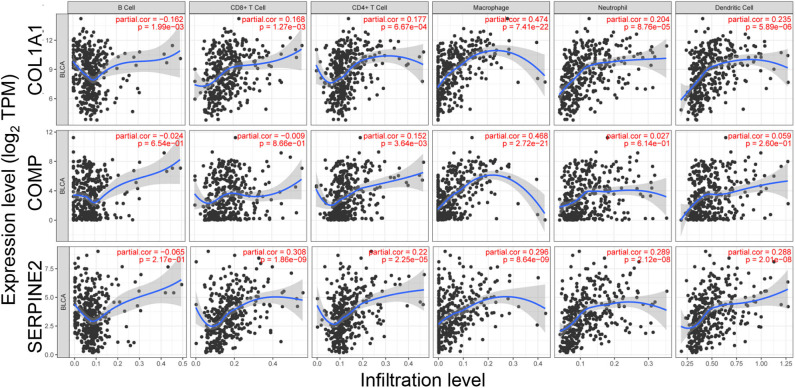
Correlation of prognostic genes' expression with immune infiltration level.

To investigate the associations between the prognostic genes and infiltrating immune cells in BC, the abundances of six immune cells were analyzed using TIMER. As presented graphically in Figure 6, the expression of COL1A1 have a positive correlation with the infiltrating levels of CD8^+^ T cells (*p* = 1.27e-03), CD4^+^ T cells (*p* = 6.67e-04), macrophages (*p* = 7.41e-22), neutrophils (*p* = 8.76e-05), and dendritic cells (*p* = 5.89e-06), while having a negative correlation with that of B cells (*p* = 1.99e-03). COMP was also positively related with infiltrating levels of CD4^+^ T cells (*p* = 3.64e-03) and macrophages (*p* = 2.72e-21; Figure 6). Moreover, the expression of SERPINE2 was positively related with the infiltrating levels of CD8^+^ T cells (*p* = 1.86e-09), CD4^+^ T cells (*p* = 2.25e-05), macrophages (*p* = 8.64e-09), neutrophils (*p* = 2.12e-08), and dendritic cells (*p* = 2.01e-08; Figure 6). These data suggested that COL1A1, COMP, and SERPINE2 might play important roles in the activation and recruitment of immune cells in BC.

## Discussion

TME is a complex system that contains fibroblasts, epithelial cells, and infiltrating immune cells, as well as extracellular matrix, which all support neoplastic proliferation, invasion, transformation, and immune tolerance. Tumor cells and infiltrating cells in the TME can produce high levels of immunosuppressive cytokines to inhibit T cell proliferation and effector function while promoting tumor development (26, 27). In addition, tumor-expressing specific proteins can suffice to induce immune cell deactivation and facilitate immune evasion (28). As the most common malignancy of the urinary system, BC has been intensely studied, but the relationship between immune microenvironment and prognosis of BC remains poorly understood. Therefore, it will be highly necessary to understand the underlying mechanism.

In this study, we first calculated immune and stromal scores of BC samples using the ESTIMATE algorithm and analyzed the relationships between these scores and clinical characteristics and prognosis of BC patients. We found that the stromal and immune scores were significantly associated with the clinical pathologic characteristics of BC (e.g., invasiveness, histological grade, and stage) and the patients' prognosis. Both immune and stromal scores of MIBC, high-grade, and later stage BC were significantly higher compared to NMIBC, low-grade, and earlier stage groups, while the immune and stromal scores were not associated with BC recurrence and progression. In addition, higher immune and stromal scores showed close associations with poorer CSS. However, there was no significant correlation between the immune/stromal scores and OS of BC patients. These results are generally consistent with previous studies (15). Our findings indicated that the TME composition could affect the clinical outcomes of BC patients.

In the TME, CAFs are able to express and secret multiple soluble factors, such as growth factors, chemokines, and extracellular matrix-related proteins. Increased secretion of these soluble factors has been proven to restrain anti-tumor immunity by blocking T cells infiltration (29) and be closely connected with poor prognosis in clinical oncology (30). Moreover, increasing research suggests that CAFs are predictors for poor prognosis in various malignancies, including BC (31). Tumor-associated endothelial cells are essential angiogenic regulators for tumor growth and are regulated by various signals from nearby infiltrating immune cells, tumor cells, and stromal cells (32). Previous researchers have found that increased macrophage presence in the TME is correlated with poor prognosis of BC patients (11). In this study, we analyzed the proportions of immune cells in the TME of the high and low score groups to explain why the outcomes of the patients with higher scores were poorer. We found that the relative abundances of CAFs, endothelial cells, and macrophages were significantly increased in both high immune and stromal score groups compared to the low score groups, whereas that of CD8^+^ T cells was significantly decreased in the high immune score group. These results suggested that the infiltrations of immune cells displaying immunosuppressive functions were increased in BC.

Next, we identified immune-related DEGs between the high and low immune/stromal score groups. A total of 104 overlapping genes were obtained for further analysis. The GO enrichment analysis showed that the immune-related DEGs were strongly involved in extracellular matrix organization, inflammatory response, and immune response that were associated with the immune microenvironment of tumors. Previous research shown that focal adhesion kinase was a crucial signaling molecule in regulating fibrotic and immunosuppressive TME and its expression was negatively associated with the infiltration of T cells (33). PI3K-Akt signaling pathway is a well-known transduction pathway involved in tumorigenesis, proliferation, and immune microenvironment (34). In this study, KEGG pathway analysis showed that enrichments of the immune-related DEGs clustered observably in focal adhesion and PI3K-Akt signaling pathway. These results indicated that the immune-related DEGs were involved in the proliferation and progression of BC by affecting the immune microenvironment.

Since LASSO is recognized as a type of penalized regression, we used LASSO regression analysis to screen covariates. With the combination of using univariate analysis and regression coefficient, we identified three prognostic immune-related genes and subsequently constructed a risk prediction model showing a great capacity for predicting CSS and OS. Patients with BC were stratified to high- and low-risk groups based on the best cutoff value of the risk score. Higher expression levels of COL1A1, COMP, and SERPINE2 were observed in the high-risk group compared with the low-risk group. Unfortunately, ~55.3% (21/38) of high-risk patients died of BC within 3 years of diagnosis, while less than 21% (9/43) died in the low-risk group. Therefore, we suggest that BC patients in the high-risk group should receive more aggressive therapy and more frequent follow-up after diagnosis.

Further survival analysis indicated that the high expression levels of COL1A1, COMP, and SERPINE2 were associated with poor CSS and OS in BC patients. These genes have been proven to be involved in proliferation, migration, development, and progression of various cancers. COL1A1 has been considered as an oncogene and promoted cancer migration and invasion by inducing epithelial-mesenchymal transition (35). Brooks et al. (36) found that the high COL1A1 expression level was associated with poor survival in patients with NMIBC, which was in line with our results. In addiation, increasing evidence confirmed that inhibition of COL1A1 could significantly suppress cancer cells proliferation, clonogenicity, and invasion, indicating that COL1A1 is a putative therapeutic target for cancer (35, 37, 38). COMP, encoding a non-collagenous extracellular matrix protein, is up-regulated in various cancers and involved in cancer cell proliferation, tumorigenesis, epithelial-mesenchymal transition, and stemness features (39–41). It has been suggested as an independent prognostic marker for breast cancer (41). Liu et al. (42) established COMP-knockout cells to detect the effects of COMP on colon cancer cells and found that knockout of COMP could suppress cells proliferation, clonogenicity, and tumor growth, while increasing sensitivity to chemotherapy. These findings indicate that COMP may be also a potential therapeutic target for cancer. Moreover, SERPINE2 contributes to enhanced invasion, metastasis, and stemness of cancer cells by remodeling the tumor matrix, and has been identified as a poor biomarker for endometrial cancer (43). SERPINE2 knockdown has been proven to inhibite tumor cell invasion and metastasis, indicating that SERPINE2 likewise can serve as a potential therapeutic target (44, 45). Nonetheless, these identified genes received less research and attention in BC. Our findings indicated that they could serve as potential prognostic markers and therapeutic target for BC.

As the primary components of the TME, immune cells play an important part in modulating tumor behavior and response to treatment (46, 47). Finally, we used the TIMER database to investigate the associations between the prognostic genes and infiltrating immune cells in BC. Our findings indicated that there is a significantly positive correlation between the expression levels of COL1A1 and SERPINE2 and the infiltrating levels of CD8^+^ T cells, CD4^+^ T cells, macrophages, neutrophils, and dendritic cells in BC. COMP was positively associated with infiltrating levels of CD4^+^ T cells and macrophages. These correlations suggested that COL1A1, COMP, and SERPINE2 might play important roles in the activation and recruitment of immune cells in BC.

## Data Availability Statement

The datasets presented in this study can be found in online repositories. The names of the repository/repositories and accession number(s) can be found in the article/Supplementary Material.

## Author Contributions

FL and YW developed the idea and designed the study. FL, HT, and ML conducted the study, collected, and analyzed the data. BL, DZ, and HZ interpreted the data. FL wrote this article. FL and ZX accomplished the interpretation of data and participated in the revision. HZ and YW were responsible for editing and submitting this manuscript. All authors approved the final version and submission.

## Conflict of Interest

The authors declare that the research was conducted in the absence of any commercial or financial relationships that could be construed as a potential conflict of interest.
