# Supplementary material for: Prognostic Value of Immune-Related Genes in the Tumor Microenvironment of Bladder Cancer
Source: Front Oncol. 2020 Jul 28;10:1302. doi: 10.3389/fonc.2020.01302 (PMC7399341; doi:10.3389/fonc.2020.01302)
Supplement: Supplementary file 1 [file Data_Sheet_1.docx]

**Supplementary Table 1.** The immune and stromal scores for 165 samples from GSE13507.

| Sample | ImmuneScore | StromalScore | Sample | ImmuneScore | StromalScore |
| --- | --- | --- | --- | --- | --- |
| GSM340605 | -259.724 | -1233.4 | GSM340688 | -379.326 | -1072.4 |
| GSM340606 | 366.8437 | 390.7677 | GSM340689 | 627.7357 | -436.877 |
| GSM340607 | 999.0014 | -517.057 | GSM340690 | 444.5078 | -533.897 |
| GSM340608 | -513.001 | -1818.93 | GSM340691 | 615.6919 | -515.126 |
| GSM340609 | -378.762 | -1316.13 | GSM340692 | -384.56 | -1158.01 |
| GSM340610 | 304.775 | -1268.23 | GSM340693 | 876.8329 | -321.047 |
| GSM340611 | -377.854 | -1411.69 | GSM340694 | 874.9214 | -667.214 |
| GSM340612 | 408.6965 | 501.6256 | GSM340695 | 104.9926 | -697.834 |
| GSM340613 | 497.5922 | -684.237 | GSM340696 | 1147.848 | -122.678 |
| GSM340614 | -550.599 | -1056.71 | GSM340697 | 1098.269 | 821.2493 |
| GSM340615 | 236.877 | -1082.8 | GSM340698 | -347.429 | -1266.1 |
| GSM340616 | 412.3775 | -701.016 | GSM340699 | 1233.972 | 455.359 |
| GSM340617 | -33.5294 | -1362.3 | GSM340700 | -13.4871 | -1198.95 |
| GSM340618 | -494.257 | -1473.78 | GSM340701 | -960.888 | -1195.68 |
| GSM340619 | -589.878 | -1387.56 | GSM340702 | 555.6309 | -431.159 |
| GSM340620 | -360.154 | -613.167 | GSM340703 | 465.8595 | -1015.54 |
| GSM340621 | -641.415 | -1522.79 | GSM340704 | -852.197 | -1384.5 |
| GSM340622 | -13.5174 | -1424.44 | GSM340705 | 1885.22 | 991.4387 |
| GSM340623 | 247.1792 | -974.095 | GSM340706 | 402.6537 | -803.352 |
| GSM340624 | -239.426 | -1311.37 | GSM340707 | -135.259 | -1588.72 |
| GSM340625 | -539.951 | -1742.43 | GSM340708 | 199.4245 | -1051.76 |
| GSM340626 | -246.004 | -1272.59 | GSM340709 | -450.057 | -502.974 |
| GSM340627 | -321.537 | -1211.45 | GSM340710 | -99.4876 | -616.51 |
| GSM340628 | 255.3435 | -944.869 | GSM340711 | -90.2729 | -460.69 |
| GSM340629 | 751.5536 | -362.276 | GSM340712 | -172.42 | -854.775 |
| GSM340630 | -517.778 | -1161.76 | GSM340713 | -222.061 | -428.182 |
| GSM340631 | -105.004 | -1493.98 | GSM340714 | 246.7153 | 191.8999 |
| GSM340632 | -265.318 | -946.68 | GSM340715 | 800.6774 | -367.899 |
| GSM340633 | -224.174 | -875.139 | GSM340716 | 34.52453 | -730.98 |
| GSM340634 | -97.9752 | -857.988 | GSM340717 | -203.001 | -1306.55 |
| GSM340635 | 141.7958 | -463.823 | GSM340718 | 381.3287 | -100.918 |
| GSM340636 | 559.0793 | -1483.14 | GSM340719 | 472.3004 | -610.935 |
| GSM340637 | 403.4413 | -962.741 | GSM340720 | 1593.213 | 1016.635 |
| GSM340638 | -187.209 | -1295.41 | GSM340721 | -325.588 | -399.203 |
| GSM340639 | -123.341 | -1033.44 | GSM340722 | 530.8494 | -610.678 |
| GSM340640 | 78.05554 | -1376.01 | GSM340723 | 247.9009 | -895.526 |
| GSM340641 | -934.612 | -1937.45 | GSM340724 | -389.985 | -1024.52 |
| GSM340642 | -146.378 | -422.51 | GSM340725 | 396.2059 | -169.912 |
| GSM340643 | -227.078 | -1555.72 | GSM340726 | 42.99964 | -947.783 |
| GSM340644 | 21.34368 | -1257.46 | GSM340727 | 676.0786 | -159.332 |
| GSM340645 | 223.1553 | -1192.55 | GSM340728 | 853.5343 | -652.389 |
| GSM340646 | -329.274 | -1100.79 | GSM340729 | -897.9 | -1811.11 |
| GSM340647 | -351.611 | -1431.15 | GSM340730 | 158.7822 | -808.324 |
| GSM340648 | -72.5576 | -642.081 | GSM340731 | 352.2669 | -773.969 |
| GSM340649 | 351.6992 | -606.614 | GSM340732 | 1345.508 | -249.497 |
| GSM340650 | 898.4807 | -274.526 | GSM340733 | 1457.928 | 304.3334 |
| GSM340651 | -106.38 | -849.778 | GSM340734 | 34.53686 | -918.882 |
| GSM340652 | -430.144 | -1544.35 | GSM340735 | -344.773 | -1213.02 |
| GSM340653 | -140.044 | -759.121 | GSM340736 | -187.589 | -849.578 |
| GSM340654 | 865.8132 | -365.95 | GSM340737 | -431.387 | -811.059 |
| GSM340655 | 62.04046 | -1033.63 | GSM340738 | 77.15164 | -1050.25 |
| GSM340656 | -17.3755 | -1328.24 | GSM340739 | 823.1883 | -501.773 |
| GSM340657 | 912.4181 | -607.808 | GSM340740 | 327.4066 | -384.878 |
| GSM340658 | -139.508 | -874.898 | GSM340741 | 651.5536 | -759.447 |
| GSM340659 | 441.4882 | -1002.66 | GSM340742 | 638.4489 | 294.3886 |
| GSM340660 | 563.0004 | 597.3406 | GSM340743 | 441.0037 | -470.42 |
| GSM340661 | -57.6617 | -975.028 | GSM340744 | 781.9983 | -359.517 |
| GSM340662 | -416.309 | -1096 | GSM340745 | 840.8938 | -1037.97 |
| GSM340663 | 153.1549 | -1002.95 | GSM340746 | 1195.34 | 1429.595 |
| GSM340664 | -464.181 | -1463.14 | GSM340747 | -309.549 | -1074.31 |
| GSM340665 | 49.83016 | -215.092 | GSM340748 | -202.164 | -1446.66 |
| GSM340666 | -238.8 | -1374.96 | GSM340749 | 891.4095 | 481.7045 |
| GSM340667 | -149.191 | -1799.33 | GSM340750 | -128.542 | -60.0308 |
| GSM340668 | -439.278 | -1939.84 | GSM340751 | 309.8843 | -701.695 |
| GSM340669 | -453.585 | -1134.43 | GSM340752 | 246.9459 | -291.586 |
| GSM340670 | 230.0612 | -465.823 | GSM340753 | -256.68 | -986.392 |
| GSM340671 | -262.572 | -1029.72 | GSM340754 | -138.394 | -982.576 |
| GSM340672 | -348.926 | -840.723 | GSM340755 | -320.656 | -1126.63 |
| GSM340673 | 341.2963 | -507.579 | GSM340756 | 51.74053 | -644.46 |
| GSM340674 | 870.4058 | 913.3797 | GSM340757 | -569.623 | -1626.11 |
| GSM340675 | 242.1947 | -1226.07 | GSM340758 | 708.7745 | 636.3531 |
| GSM340676 | 10.20786 | -922.041 | GSM340759 | 758.4806 | 750.4867 |
| GSM340677 | 375.5437 | -756.158 | GSM340760 | -345.775 | -713.747 |
| GSM340678 | 107.2345 | -290.265 | GSM340761 | 714.2401 | 1022.497 |
| GSM340679 | 213.1233 | -1169.24 | GSM340762 | 318.5276 | -602.535 |
| GSM340680 | -230.982 | -1079.97 | GSM340763 | 949.6536 | -171.478 |
| GSM340681 | 151.6513 | -948.831 | GSM340764 | -264.977 | -748.094 |
| GSM340682 | -246.353 | -1341.55 | GSM340765 | -131.803 | -1103.79 |
| GSM340683 | -360.542 | -1119.32 | GSM340766 | 2023.121 | -4.52037 |
| GSM340684 | 568.9812 | -861.975 | GSM340767 | 128.3669 | -1228.83 |
| GSM340685 | 18.18947 | -943.611 | GSM340768 | -362.594 | -914.869 |
| GSM340686 | 8.95464 | -895.979 | GSM340769 | 1418.1 | 1029.412 |
| GSM340687 | 1157.564 | 463.9887 |  |  |  |

**Supplementary Table 2.** Univariate Cox regression analysis of prognostic factors for CSS.

| Gene | HR (95%CI) | p value | Gene | HR (95%CI) | p value |
| --- | --- | --- | --- | --- | --- |
| COMP | 1.762(1.399-2.219) | 1.52E-06 | OLR1 | 1.399(1.077-1.817) | 0.012 |
| COL1A1 | 1.652(1.304-2.092) | 3.12E-05 | LY96 | 1.454(1.077-1.963) | 0.014 |
| SERPINE2 | 1.534(1.251-1.881) | 3.86E-05 | PDPN | 1.474(1.077-2.017) | 0.015 |
| COL5A1 | 1.706(1.285-2.265) | 2.22E-04 | IL8 | 1.237(1.041-1.471) | 0.016 |
| AEBP1 | 1.494(1.202-1.858) | 2.99E-04 | ACTN1 | 1.430(1.069-1.914) | 0.016 |
| COL8A1 | 1.607(1.242-2.080) | 3.10E-04 | DSG3 | 1.311(1.051-1.635) | 0.017 |
| SULF1 | 1.530(1.212-1.931) | 3.43E-04 | COL1A2 | 1.355(1.054-1.742) | 0.018 |
| VCAN | 1.489(1.183-1.873) | 6.81E-04 | PRKCDBP | 1.389(1.058-1.822) | 0.018 |
| COL18A1 | 1.687(1.243-2.290) | 7.95E-04 | CCL2 | 1.332(1.047-1.694) | 0.020 |
| TNC | 1.466(1.141-1.883) | 0.003 | COL6A3 | 1.337(1.047-1.708) | 0.020 |
| COL3A1 | 1.470(1.141-1.893) | 0.003 | CYR61 | 1.316(1.043-1.660) | 0.021 |
| CD248 | 1.630(1.168-2.274) | 0.004 | TGFBI | 1.293(1.040-1.608) | 0.021 |
| COL16A1 | 1.409(1.111-1.786) | 0.005 | AIF1 | 1.378(1.048-1.811) | 0.022 |
| COL15A1 | 1.565(1.143-2.143) | 0.005 | CTGF | 1.242(1.032-1.494) | 0.022 |
| F3 | 1.522(1.133-2.043) | 0.005 | SERPINE1 | 1.306(1.037-1.644) | 0.023 |
| ITGA5 | 1.370(1.098-1.709) | 0.005 | TGFB3 | 1.314(1.028-1.678) | 0.029 |
| S100A7 | 1.295(1.079-1.555) | 0.006 | GAS1 | 1.389(1.031-1.870) | 0.031 |
| PDGFRB | 1.570(1.139-2.164) | 0.006 | FLNA | 1.300(1.022-1.653) | 0.033 |
| SPP1 | 1.298(1.077-1.563) | 0.006 | CDH11 | 1.378(1.025-1.853) | 0.034 |
| DIO2 | 1.522(1.125-2.059) | 0.006 | TPM1 | 1.219(1.003-1.481) | 0.046 |
| GREM1 | 1.463(1.104-1.939) | 0.008 | C1S | 1.266(1.002-1.600) | 0.048 |
| TSC22D3 | 1.441(1.098-1.890) | 0.008 | LGALS1 | 1.280(1.002-1.636) | 0.048 |
| IER3 | 1.381(1.079-1.768) | 0.010 |  |  |  |

CSS, cancer-specific survival; HR, Hazard ratio; 95%CI, 95% confidence interval


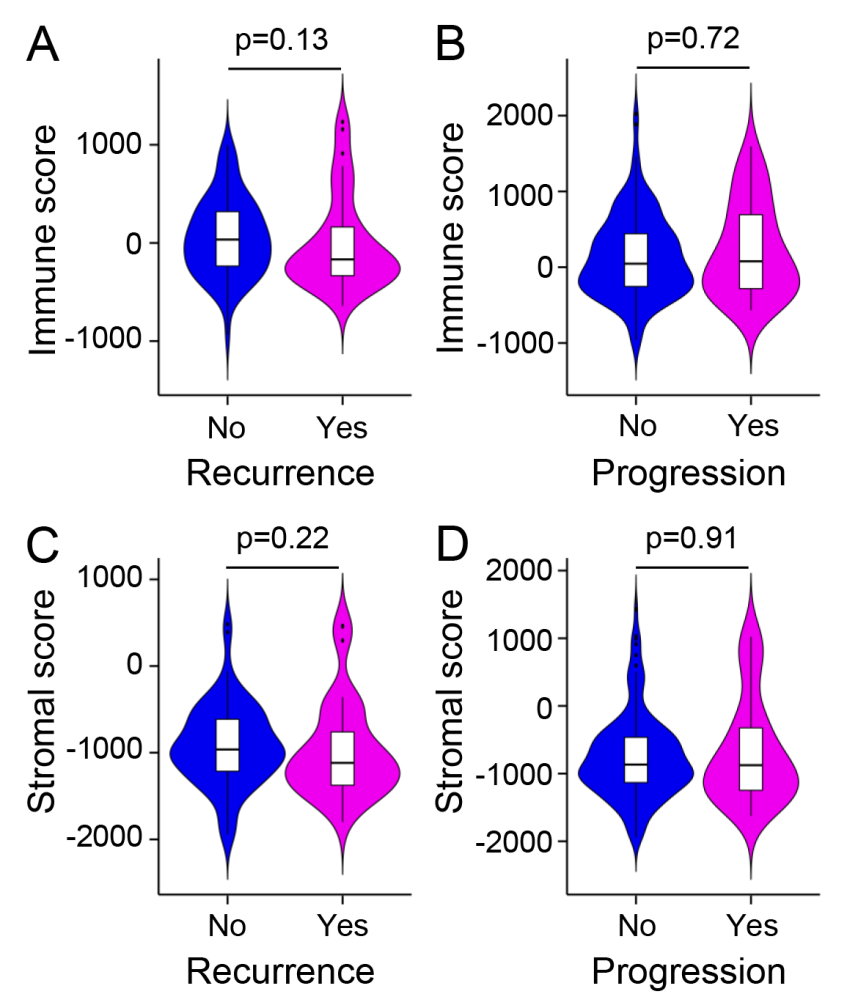


**Supplementary Figure 1.** Relationship between immune and stromal scores and BC clinical characteristics. **(A)** Distributions of immune scores between BC recurrence and non-recurrence groups. **(B)** Distributions of immune scores between BC progression and non-progression groups. **(C)** Distributions of stromal scores between BC recurrence and non-recurrence groups. **(D)** Distributions of stromal scores between BC progression and non-progression groups. BC, bladder cancer.


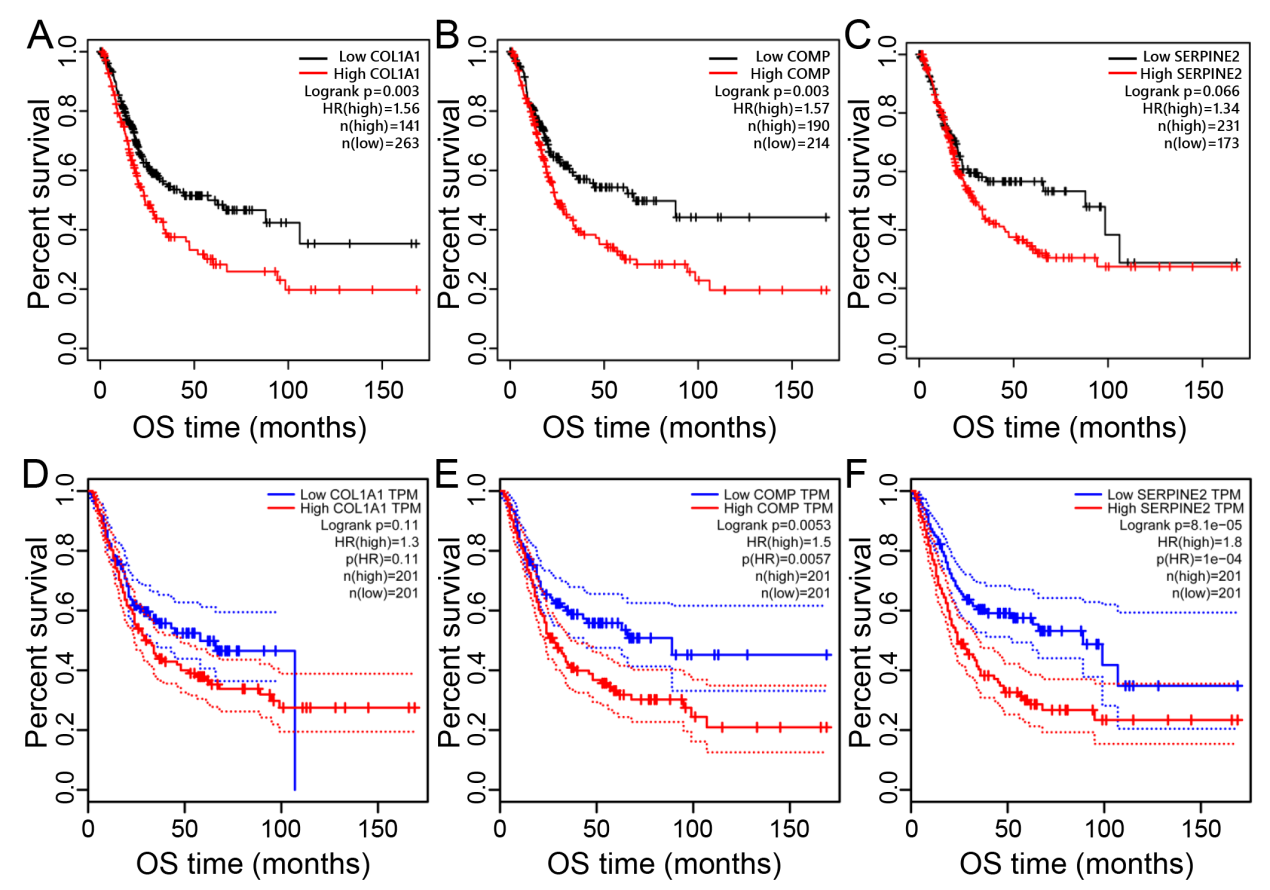


**Supplementary Figure 2.** Correlation of the prognostic genes with overall survival of BC patients. **(A-C)** Kaplan-Meier curves of OS for patients grouped by expression levels of COL1A1, COMP, and SERPINE2, which were obtained from the Kaplan-Meier Plotter database. (D-F) Kaplan-Meier curves of OS for patients grouped by expression levels of COL1A1, COMP, and SERPINE2, which were obtained from the GEPIA database. BC, bladder cancer; OS, overall survival; GEPIA, Gene Expression Profiling Interactive Analysis.


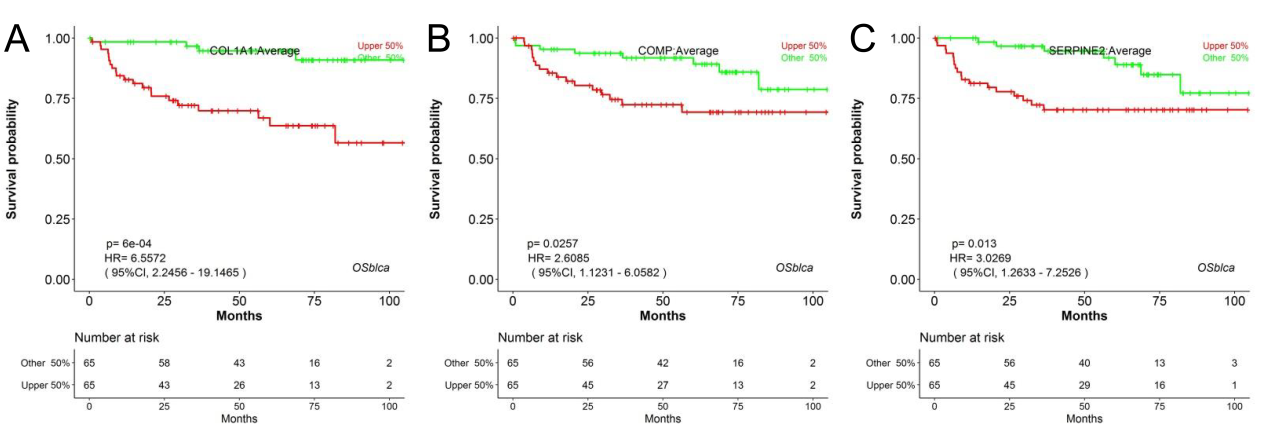


**Supplementary Figure 3.** Correlation of the prognostic genes with overall survival of BC patients from the dataset GSE32548. **(A-C)** Kaplan-Meier curves of OS for patients grouped by expression levels of COL1A1, COMP, and SERPINE2, which were obtained using the online survival analysis tool OSblca. BC, bladder cancer; OS, overall survival.
